# Investigating the Linkages between BMI, Body Image, and SATAQ among Young Asian Females

**DOI:** 10.3390/ijerph18147460

**Published:** 2021-07-13

**Authors:** Jane Lu Hsu, Rainbow Tsai-Ling Hung, Melchior Antoine

**Affiliations:** Department of Marketing, National Chung Hsing University, Taichung 402204, Taiwan; rainbowww5588@yahoo.com.tw (R.T.-L.H.); melchiorantoine1984@gmail.com (M.A.)

**Keywords:** sociocultural attitudes, body mass index, body image, Asia

## Abstract

Sociocultural attitudes toward appearance and its linkage to body mass index (BMI) and body image is a relationship worth studying, especially in Asia, where the idealization of thinness can be prominent. The Sociocultural Attitudes Toward Appearance Questionnaire (SATAQ) developed by Heinberg et al. in 1995 was used in this study to examine whether these beliefs have been internalized. Furthermore, the Body Esteem Scale (BES) was used to quantify body image. The formal in-person survey was administered in Taipei, Taiwan. There were 301 valid samples out of 330 surveyed respondents. To enhance the representativeness of the samples, a stratified sampling technique was applied to generate survey data with valid samples following gender and age distributions of the population between the ages of 14 and 28. The internalization dimension represented how strongly the respondents considered the importance of the socially accepted concept of appearance based on personal perceived social norms. The other dimension, awareness, represented the sociocultural pressures from the outside world, such as from the image of slimness illustrated in the media or group, in comparison to personal beliefs. Our results demonstrated that respondents with above-normal BMIs internalized the socially preferred standards of thinness more than respondents with normal or under-normal BMIs. Furthermore, results also revealed, overall, a negative relationship between SATAQ and body image and between body image and BMI. The study highlights the need to emphasize “fitness over thinness” to help improve negative body image among young Asian females.

## 1. Introduction

Sociocultural attitudes toward appearance in young females are often heavily influenced by social norms that are amplified by social networks, peer groups, and fashion marketing in both traditional and social media targeted towards the youth [1]. In terms of appearance, the importance of thinness for the female body is deeply rooted in modern societies, and the internalization of sociocultural attitudes may explain the drive for slimness without health considerations [2]. Martin and Kennedy state that, according to social comparison theory, the process of stereotyping thinness occurs when women consciously or subconsciously compare their own bodies to the physical traits of models and actresses, in which slimness is strongly emphasized [3]. However, studies continue to show that media, in particular social media such as Instagram, negatively influences young women’s body image [4,5]. In particular, the ideal of thinness and the accompanying effects that it has on young females’ body image are especially prominent in Asian contexts, where the ideal of thinness is emphasized quite heavily [6,7,8,9]. In this study, we sought to examine the body image and sociocultural attitudes among a sample of women in an Asian country. We also attempted to determine the relationship between body mass index (BMI) and body image, since BMI is often used as an indicator of healthy body weight [10], as well as the relationship between sociocultural attitudes and body image, as studies have revealed a relationship between these two factors [9,11,12].

### 1.1. Sociocultural Attitudes toward Appearance

Sociocultural attitudes tend to significantly influence body image among individuals [6]. Furthermore, research has suggested that young women tend to internalize the ideal of thinness due to social pressures, with greater idealization leading to a more negative self-image [2,6,7,8,9]. The Sociocultural Attitudes Toward Appearance Questionnaire (SATAQ) was developed to evaluate women’s recognition and acceptance of social standards of appearance [13]. The main purpose of the questionnaire is to measure the main social beliefs about thinness and whether these beliefs have been internalized. The questionnaire consists of a total of 14 items or statements to be graded on a scale ranging from 1 to 6 in terms of agreement.

### 1.2. Body Image and Body Mass Index

Body image generally refers to an individual’s mental attitudes toward themselves, and it usually involves body esteem and body dissatisfaction [4,14,15]. It is also commonly measured with the Body Esteem Scale (BES) scale (Mendelson et al., 1996). Studies have shown that a number of factors affect body image [14,15]. In a seminal study, Mendelson et al. indicated that there was a negative relationship with weight as measured by BMI and body image, which means that heavier individuals tended to have lower scores for body image [14]. Other studies have suggested a similar relationship [10,16,17,18].

Body mass index (BMI), which is weight in kilograms divided by height in squared meters is commonly used as an objective scientific measure of body weight [10] and has been suggested by the World Health Organization (WHO) as a measure for an indication of potential health concerns once the measure goes beyond suggested cut-off points [19]. The current cut-off points recommended by the WHO are as follows: larger than or equal to 25 kg/m^2^ for being overweight and larger than or equal to 30 kg/m^2^ for obesity [19]. However, researchers have indicated the need for considerations of the differences in bodyweight between Asians and Western populations [19,20,21].

Taking all this into consideration, the current study employs the BMI classification from the Department of Health, Executive Yuan, Taiwan, as listed in Table 1, where BMI cut-off points for Chinese/Taiwanese adults with a BMI < 18.5 are considered as underweight, with 18.5 ≤ BMI ≤ 24 being normal, and BMI > 24 being categorized as above-normal [22].

### 1.3. Hypotheses

The literature suggests a significantly negative relationship between BMI and body image [2,6,7,8,9]. Furthermore, it is also expected that individuals who internalize the sociocultural idea of thinness would be likelier to have a negative self-image, as has been shown in the literature [10,16], and that heavier individuals were also more likely to internalize sociocultural attitudes [7,9].

**Figure 1:**
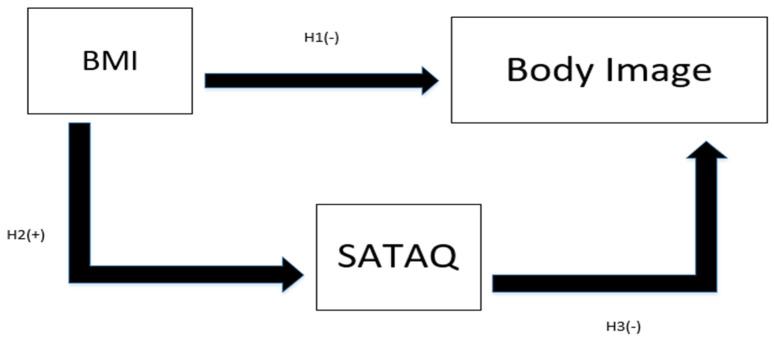
Conceptual Framework of the Study.

In consideration of these relationships, the specific objective of this study is to reveal sociocultural attitudes toward appearance and body image for young Asian females according to BMI. Furthermore, the hypotheses of the study are as follows and the study framework considering these hypotheses is depicted in Figure 1.

**Hypothesis** **1** **(H1).** *There is a negative relationship between BMI and body image*.

**Hypothesis** **2** **(H2).** *There is a positive relationship between BMI and sociocultural attitudes toward appearance*.

**Hypothesis** **3** **(H3).** *There is a negative relationship between sociocultural attitude toward appearance and body image*.

In light of this discourse, the overall goal of this study is to provide useful information in terms of understanding the sociocultural attitudes of young Taiwanese females.

## 2. Materials and Methods

### 2.1. Instruments

The Sociocultural Attitudes Toward Appearance Questionnaire (SATAQ) [13] is a 14-item measure of the extent to which individuals regard main social beliefs about thinness and whether these beliefs have been internalized. The instrument has displayed satisfactory reliability and validity in children and adults [13]. The items included in SATAQ are statements such as “Women who appear in TV shows and movies project the type of appearance that I see as my goal (Item 1)”, “In our society, fat people are not regarded as unattractive (Item 6)”, and “I wish I looked like a swimsuit model (Item 13)”. The full list of items included in the table can be found later in the article. The instrument is based on a Likert-type scale, and the items are ranked from 1 to 6, with 1 representing “strongly disagree” and 6 “strongly agree.” The validity of SATAQ for Taiwan, especially for the Awareness and Internalization dimension, has already been established by Chen et al. [23]. Chen et al. reported internal consistency values of 0.82 and 0.72 for the SATAQ Awareness and Internalization dimensions, respectively.

The measure for body image was the Body Esteem Scale (BES) [14]. The BES is a 20-item measurement to reveal whether a person has positive feelings and beliefs about his or her own body shape. The instrument has displayed acceptable reliability and validity for both children and adults [14]. The items in the BES scale include positive statements on body satisfaction such as “I like what I look like in pictures” and “Kids my own age like my looks”, and negative statements on body satisfaction such as “Most people have a nicer body than I do” and “My looks upset me”. The full list of items for the BES scale is provided later in the article. The original versions of SATAQ and the BES scale were used, and for the Chinese-speaking population who participated in the study, the items in the scale were translated and reverse translated to ensure accuracy.

### 2.2. Data Collection Procedure

Respondents’ height and weight (for BMI calculations), age, educational level, occupation, and monthly personal income were queried in the questionnaire in addition to sociocultural attitudes toward appearance. A trial survey was administered prior to the formal survey and included 15 participants. The questionnaire was modified based on suggestions provided by professionals and respondents participating in the trial survey. The formal in-person survey was administered in the Taipei metropolitan area and 330 questionnaires were distributed. All respondents who participated had to provide informed consent before participation, and for minors, we obtained consent from their parents. The questionnaire was completely anonymous, and there was no need to apply for ethical approval. The total number of valid samples was 301 out of 330 surveyed respondents. To enhance the representativeness of the sample, a stratified sampling technique was applied to generate survey data with valid samples following gender and age distributions of the population between the ages of 14 and 28.

Descriptive statistics, factor analysis, and multivariate analysis of variance (MANOVA) were used in analyzing the data. The purpose of factor analysis was to create a new set of uncorrelated variables from a set of corrected variables and attempt to explain the correlations between the original variables [24]. In this study, the principal factoring method was used with varimax rotation in the factor analysis. Cronbach’s alpha coefficients were calculated to measure the internal consistency of multi-item scales. MANOVA was applied to examine statistical differences among the different BMI segments in terms of sociocultural attitudes toward appearance.

## 3. Results

The objective of this exploratory study is to reveal sociocultural attitudes toward appearance and its linkage to BMI for young females in Asia, in particular, Taiwan. Based on the survey results, the average age of respondents was 21.2 years old. The average height of respondents was 160.9 cm, and the weight of respondents was averaged at 52.5 kg, with an average BMI of 20.3. Less than half of the respondents had a college educational level (41.3%), and slightly more than 30% of respondents had been to graduate school. The average monthly personal expenditure of surveyed respondents was USD 222.3. The demographic characteristics of respondents are listed in Table 2.

### 3.1. Structural Equation Modelling Results

**Figure 2:**
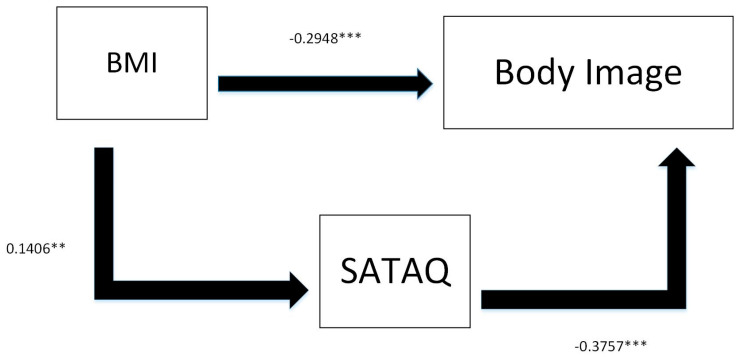
The SEM Model Results. ** denotes values significant at the 5% significance level; *** denotes values significant at the 1% level.

Structural equation model (SEM) testing was conducted using SAS, and the results revealed that, according to the estimate values, the model was a good fit. The results are depicted in Table 3 and Figure 2.

### 3.2. Sociocultural Attitudes toward Appearance

Factor analysis was used in this study to reveal various dimensions of sociocultural attitudes toward the appearance of respondents. A principal factorizing procedure with varimax rotation was applied. Fourteen sociocultural attitudes toward appearance items were factorized into two dimensions based on Kaiser’s criterion of selecting a number of dimensions with eigenvalues greater than one. Four statements out of 14 sociocultural attitudes toward appearance were dropped in the factor analysis due to low loading on underlying dimensions. The sociocultural attitudes toward appearance with those four items deleted were factorized again, which then revealed a two-factor solution, *internalization* and *awareness* (Table 4). The total variance explained was 53.76%. The internalization dimension represented how strong the respondents would consider the importance of the socially accepted concept of appearance based on personal perceived social norms. The other dimension, awareness, represented the sociocultural pressures from the outside world, such as from the image of slimness illustrated in the media or the group in comparison, on personal beliefs. Cronbach’s alpha coefficients were used to measure the internal consistency of the factorized dimensions. In the two dimensions for sociocultural attitudes toward appearance, Cronbach’s alpha coefficient for the internalization dimension was 0.83, and 0.59 for its awareness dimension.

### 3.3. Body Image

The measure of body image used in this study was the BES [14]. Principal factorizing procedure with varimax rotation was applied. After factorizing the BES statements, three statements were dropped: “There are lots of things I’d change about my looks if I could”, “I’m proud of my body”, and “I think I have a good body”. The factor analysis was applied to the BES statements again with the removal of these three statements. The results revealed that there were three essential dimensions for body image, including self-perception of look, self-perception of weight, and self-perception of anxiety (Table 5).

The first factor, self-perception of look, explained the attitudes toward personal appearance and body shape. The dimension of self-perception of look included seven items and accounted for 33% of the total variance, with the Cronbach’s alpha coefficient equal to 0.81. The second factor, self-perception of weight, explained feelings about individuals’ personal weight. The dimension of self-perception of weight consisted of five items and accounted for 13% of the total variance with the Cronbach’s alpha equal to 0.77. The third factor, self-perception of anxiety, indicated pressure from the outside world regarding personal look or body image. The dimension of self-perception of anxiety consisted of five items and accounted for 9% of the total variance with the Cronbach’s alpha equal to 0.74. The total cumulative variance explained in the three dimensions was 54.98%.

### 3.4. Linkage between BMI, Body Image, and Sociocultural Attitudes toward Appearance

The cut-off points suggested by Zhou and from the Department of Health, Executive Yuan, Taiwan [20,22], were used to segment respondents into various BMI levels. Respondents were categorized into the following segments: BMI < 18.5, under-normal; 18.5 ≤ BMI ≤ 24, normal; and BMI > 24 above-normal. Slightly over one-fourth of respondents were in the above-normal segment. Most respondents fell within the range for normal BMI levels, and less than 10% of them were in the under-normal segment.

Two dimensions of sociocultural attitudes toward appearance, internalization and awareness, were used to examine the linkage with various BMI levels. MANOVA was used to examine overall statistical differences in these two dimensions of sociocultural attitudes toward appearance. As indicated in Table 6, young Taiwanese females in differing BMI segments differed significantly when considering both dimensions of sociocultural attitudes toward appearance. Internalization, the dimension indicating how strongly respondents valued the standards of socially preferred appearance, was significant for those who worried about how others saw them in terms of their appearance. Results of Scheffe’s comparisons confirmed that respondents with above-normal BMIs internalized the socially preferred standards of thinness more than respondents with normal or under-normal BMIs. Women appeared to have intentions of altering themselves in accordance with current societal standards. Findings in this study support the argument that the internalization of societal pressures regarding appearance is a key feature of body dissatisfaction [2,6,7,8,9].

Awareness, the other dimension of sociocultural attitudes toward appearance, indicates recognition of the sociocultural pressures from the outside world, such as the media or group in comparison. The awareness dimension displayed no significant differences among individuals in the different BMI segments. This result indicates that internalization has a stronger influence than awareness on attitudes toward appearance for young Taiwanese females, especially for those with BMIs above the normal range. Overweight young females may feel uncomfortable with their appearance because they heavily consider female societal standards.

Using the Pearson correlation coefficients, a significant negative correlation between SATAQ and body image (γ = −0.42, *p* < 0.01) was revealed. The sociocultural attitudes towards appearance were negatively correlated with body image as perceived by young Taiwanese females, as has been reported for other populations in previous research [2,6,7,8,9]. Those who were more concerned with appearances according to social norms were less confident about their body image. Self-esteem was negatively affected by the perceived importance of appearances. Hence, H1 of this study was supported (Figure 1).

The linkage of body image and BMI among young Taiwanese female consumers was negative as indicated in Table 7 (γ = −0.13, *p* = 0.02). Young Taiwanese females with high BMI levels were less confident in their body shapes. Hence, H2 of this study was also supported (Figure 1).

The overall statistical difference of the three dimensions of body image for various levels of BMI was significant, as indicated in the MANOVA F testing results. Testing results of ANOVA further revealed BMI groups differed significantly in terms of the following dimensions: self-perception of look (*p* = 0.01) and self-perception of weight (*p* < 0.01); however, there were no significant differences for the self-perception of anxiety dimension (*p* = 0.23) (Table 8). Testing results of Scheffe’s comparisons confirmed that female respondents with normal BMI were more concerned about personal appearance and body shapes. Female respondents with under-normal BMI worried about self-perception of weight than respondents of normal or overweight BMIs. In short, results suggest that thin females in Taiwan tended to display more positive self-perception of their weight, which was in line with previous research on other populations, which suggested that heavier individuals tended to have a more negative self-image [16,18]. The results of Scheffe’s comparison indicated the emphasis on self-perception of look for young Taiwanese female consumers with normal BMI.

## 4. Discussion

The objective of this exploratory study was to reveal the linkage between sociocultural attitudes toward appearance and body image and its linkage to BMI for young females in Taiwan. Slightly more than one-fourth of respondents were in the above-normal segment, with less than 10% of them in the under-normal segment. Two dimensions, internalization and awareness, were extracted from the statements of sociocultural attitudes toward appearance to examine its relationship with BMIs for respondents. Findings in this study indicated that young Taiwanese females with above-normal BMIs internalized sociocultural attitudes toward appearance in slimness. This result was in line with prior studies that have shown a positive link between BMI and thin-ideal internalization and awareness [7,9].

Furthermore, the relationships between SATAQ, body image, and BMI were also tested. Overall, results revealed a negative correlation between SATAQ and body image (*p* = 0.04) which supports H3, and a positive relationship between SATAQ and BMI, which supports H2, as shown in Table 7. Furthermore, there was a negative relationship between body image and BMI in terms of self-perception of look and self-perception of weight (see Table 8), which meant that individuals with lower BMIs tended to have a better body image, which supported H1.

In terms of body image, results also confirmed that respondents with under-normal BMI tended to have significantly higher self-perception of weight compared to those with normal and above-normal BMIs, respectively; moreover, respondents with under-normal BMI were less worried about self-perception of look, as shown in Table 8. In short, female respondents with under-normal BMIs worried about self-perception of weight more than respondents with normal or overweight BMIs. Such results can be explained by the fact that respondents with above-normal BMIs may be more influenced by the socially preferred standards of thinness, as indicated in the dimension of internalization, than respondents with normal or under-normal BMIs.

### Implications

The linkage between sociocultural attitudes toward appearance and BMI levels has not been exclusively examined in the literature. In this study, results demonstrated that sociocultural attitudes towards appearance varied with BMI levels in terms of internalizing socially preferred standards of thinness or from awareness of thinness based on the image of slimness emphasized in the media. Based on the results, the following conclusions can be made: (1) The socially preferred standards of thinness are more internalized for respondents with above-normal BMIs than for respondents with normal or under-normal BMIs, (2) overweight young Taiwanese female consumers feel more uncomfortable with look and body shape because they take societal standards of female thinness more seriously.

Self-perceptions regarding body image are influenced by social norms, and Asian females appear to be largely affected by slimness stereotypes [7,8]. Solutions to these negative attitudes among females with above-normal BMIs can be formulated. For example, there should be an emphasis on health standards over appearance; that is, “fitness over slimness”. The focus should be on weight loss as a means to achieve fitness as opposed to achieving thinness. Such values, however, should be introduced and encouraged through specially developed intervention programs for adolescents, as have been carried out before [25]. Dunstan et al. [26] used the Happy Being Me manualized intervention to improve body image among both boys and girls, whereas Castillo et al. [25] used specially designed workshops for university students. It is crucial that these programs are introduced at an early stage (adolescence and young adulthood), as attitudes towards body image may crystallize in later life. In addition, public media campaigns that encourage body positivity in young women can also be undertaken and encouraged. Fashion apparel companies can work together with public agencies to encourage such attitudes or even design fashion that appeals to women with lower BMI.

The relevance of this study lies in its identification of the linkages between BMI, body image, and sociocultural attitudes toward appearance among young Asian females. Internalization of the ideal of thinness among young females, especially in Asia, is common, as shown in the literature [2,6,7,8,9]. Therefore, there is a need to emphasize fitness over thinness to help improve body image among this population.

## 5. Conclusions

This study had a few limitations. We did not consider other factors that may influence sociocultural attitudes, such as income and educational level. Future studies should consider the effect of factors such as gender, income, education, etcetera, on sociocultural attitudes. Secondly, only Taiwanese females were sampled for this study. To make results more generalizable to Asian societies, future studies should incorporate a broader range of young Asian females from other Asian countries, such as Japan, Korea, Indonesia, and Malaysia, for cross-country or cross-cultural comparisons.

The study revealed that Asian females with higher BMIs tended to have a more negative body image. It also demonstrated that greater internalization and awareness in terms of sociocultural attitudes towards appearance was negatively related to body image and heavier bodyweight according to BMI. We propose emphasizing health over appearance or “fitness over thinness” as a solution to negative body image and internalization of socially desirable standards of appearance. Intervention programs in high school and among university students may help in achieving this goal. In addition, public education campaigns on the part of state agencies and fashion companies may also help in that regard. In particular, fashion companies should see this as an opportunity to cater to women with body image issues by promoting clothing that is fashionable, while catering to body types that do not fit the thin ideal.

## Figures and Tables

**Table 1:** Cut-off points of BMI from the World Health Organization (BMI = kg/m^2^).

**World Health Organization**	
**Classification**	**BMI**
Underweight	BMI < 18.5
Normal range	18.5 ≤ BMI < 24.9
Pre-obesity	25.0 ≤ BMI < 29.9
Obese class I	30.0 ≤ BMI < 34.9
Obese class II	35.0≤ BMI < 39.9
Obese class III	BMI ≥ 40.0
**Department of Health, Taiwan**	
**Classification**	**BMI**
Underweight	BMI < 18.5
Normal range	18.5 ≤ BMI < 24.0
Overweight	24.0 ≤ BMI < 27.0
Obese class I	27.0 ≤ BMI < 30.0
Obese class II	30.0 ≤ BMI < 35.0
Obese class III	BMI ≥ 35.0

**Table 2:** Demographic Characteristics of Respondents.

	Respondents (*N* = 301)
Average age (years)	21.2
Average height (cm)	160.9
Average weight (kg)	52.5
Body Mass Index	20.3
Educational level (%)	
Elementary school	0.3
Junior high school	11.0
Senior high school	15.7
College	41.3
Graduate school	31.7
Occupation (%)	
Government	3.7
Farmer	0.0
Manufacturing	1.3
Business	2.0
Service	9.3
Housewives	0.0
Students	78.4
Others	5.3
Monthly personal expenditures (USD)	222.3

Note: Monthly personal expenditures were converted into USD.

**Table 3:** Results of the SEM Model.

Variable	Estimate	Standard Error	t	*p*-Value
BMI → Body Image	−0.2948 ***	0.0486	−6.0660	<0.0001
BMI → SATAQ	0.1406 **	0.0568	2.4754	0.0133
SATAQ → Body Image	−0.3757 ***	0.0472	−7.9597	<0.0001
Direct Effect (BMI → Body Image) − 0.2948				
Indirect Effect (BMI → Body Image) − 0.0528				

Note. ** denotes values significant at the 5% significance level; *** denotes values significant at the 1% level.

**Table 4:** Factors for Sociocultural Attitudes Toward Appearance.

Factors		Loading	Variance Explained	Cronbach’s Alpha
Factor 1: Internalization			0.38	0.83
3	Music videos that show thin women make me wish that I were thin.	0.79		
13	I wish I looked like a swimsuit model	0.79		
7	Photographs of thin women make me wish that I were thin.	0.72		
5	I tend to compare my body to people in magazines and on TV.	0.72		
2	I believe that clothes look better on thin models	0.65		
14	I often read magazines like Cosmopolitan, Vogue, and Glamour and compare my appearance to the models.	0.61		
4	I do not wish to look like the models in the magazines.	0.59		
1	Women who appear in TV shows and movies project the type of appearance that I see as my goal.	0.58		
Factor 2: Awareness			0.16	0.59
10	Most people do not believe that the thinner you are, the better you look.	0.79		
12	In today’s society, it’s not important to always look attractive.	0.78		

Note: Cumulative variance explained is 53.76%. Items 6, 8, 9 and 11 were dropped from the factor analysis.

**Table 5:** Factor Loadings of Body Image Dimensions.

Factors		Loading	Variance Explained	Cronbach’s Alpha
Factor 1: Self-perception of Look.			0.33	0.81
2	Kids my own age like my looks.	0.74		
3	I’m pretty happy about the way I look.	0.70		
15	I’m looking as nice as I’d like to.	0.67		
6	I like what I see when I look in the mirror.	0.65		
1	I like what I look like in pictures.	0.64		
18	I’m as nice looking as most people.	0.56		
19	My parents like my looks.	0.50		
Factor 2: Self-perception of Weight			0.13	0.77
10	I really like what I weigh.	0.86		
7	I wish I were thinner.	0.82		
5	My weight makes me happy.	0.74		
4	Most people have a nicer body than I do.	0.60		
11	I wish I looked better.	0.56		
Factor 3: Self-perception of Anxiety			0.09	0.74
17	My looks upset me.	0.76		
16	I often wish I looked like someone else.	0.71		
12	I often feel ashamed of how I look.	0.69		
20	I worry about the way I look.	0.67		
13	Other people make fun of the way I look.	0.64		

Note: Cumulative variance explained is 54.98%. Item 8, 9, and 14 were dropped from the factor analysis.

**Table 6:** BMI and its Linkage with Sociocultural Attitudes Toward Appearance.

Sociocultural Attitudes Towards Appearance	BMI			Test Statistics	Scheffe’s Comparisons
	(1) Above-normal	(2) Normal	(3) Under-Normal		
Internalization	0.22	0.01	−0.23	*F* = 3.82 **	(1,2) > (2,3)
Awareness	0.12	−0.04	−0.03	*F* = 0.65	(1,2,3)
Wilks’ Lambda = 0.9706				*F* = 2.22 *	

Note: * indicates significance at 10% significance level; ** indicates significance at 5% significance level.

**Table 7:** Correlation Coefficient among SATAQ, Body Image, and BMI.

Items	SATAQ	Body Image	BMI
SATAQ	1.00	−0.42 *** p < 0.01	0.14 ** p = 0.02
Body Image		1.00	−0.13 ** p = 0.02
BMI			1.00

Note: ** indicates significance at 5% significance level; *** indicates significance at 1% significance level.

**Table 8:** Segments of BMI and Body Image of Respondents.

	(1) Above-Normal	(2) Normal	(3) Under-Normal	Test Statistics	*p*-Value	Scheffe’s Comparison
**Body Image**						
self-perception of look	−0.25	0.19	−0.14	*F* = 6.07 ***	0.01	(2,3) > (3,1)
self-perception of weight	−0.73	0.01	0.72	*F* = 52.70 ***	<0.01	(3) > (2) > (1)
self-perception of anxiety	−0.07	0.10	−0.12	*F* = 1.47	0.23	(2,1,3)
	Wilks’ Lambda = 0.70			*F* = 19.07 ***	<0.01	

Note: *** indicates significance at 1 percent significance level.

## Data Availability

Data sharing is applicable upon request.
